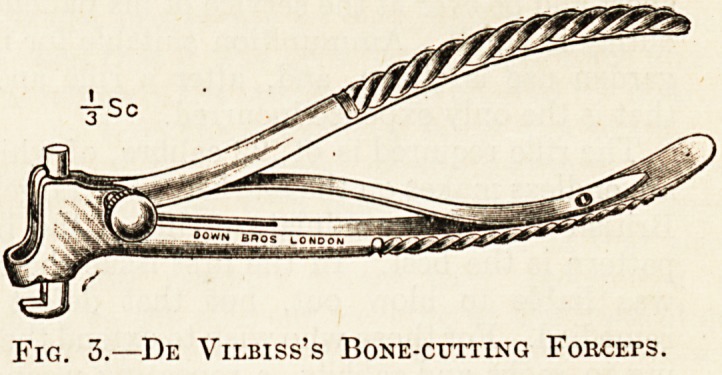# Concerning Operations for Otorrhœa

**Published:** 1908-02-01

**Authors:** 


### Otology.

### CONCERNING OPERATIONS FOR OTORRHŒA.

### III. WITH LATERAL SINUS THROMBOSIS (*concluded*).

Cases of lateral sinus thrombosis represent the chief emergencies in major aural surgery. Action must be prompt to overtake the spread of sepsis. Stage 1: In the first place, it is necessary to perform the complete radical tympano-mastoid operation. The roof and posterior wall of the mastoid cavity must be examined and removed, and the condition of the dura mater of the middle and posterior cranial fossæ ascertained to seek the path of infection. 2. Examination of the sigmoid portion of the lateral sinus. 3. Exposure of the sinus away from the infection towards the torcular. 4. Obliteration of the sinus above the infection. 5. Exposure and ligature of the internal jugular vein below the infection. 6. Removal of all septic material from the lateral sinus and its vicinity from the jugular bulb and from the jugular vein. 7. Provision of drainage. 8. After-treatment: maintenance of drainage; serum therapy.

The trephine and saw can be regarded as obsolete for these operations. The best kinds of instruments to use are the gouges, various bone-cutting forceps, and the cross-handled curette as made originally by Maw for St. Bartholomew's Hospital aural department. (The later instrument was illustrated in a recent issue of The Hospital in an article "Concerning Extra-dural Abscess.") The most useful patterns of bone forceps are Horsley's and Jansen's bone-nibbling forceps and De Vilbiss's bone-cutting forceps. In addition, the other usual instruments for the radical mastoid operation, as well as a separate set of instruments for ligaturing the internal jugular vein, will be required.

The usual post-aural incision is made. The bone beneath a superficial mastoid abscess, which may have to be opened by the incision for the radical operation, should be carefully examined with the probe for any soft track leading either to the mastoid cells, or direct from the mastoid surface, to the posterior cranial cavity. Such track, when present, should be opened up. An extra-dural abscess is present in the majority of cases, especially when the osseous plate between the sinus and antrum or cells is intact. The normal sinus is blue, elastic, pulsatile, and bleeds profusely when punctured with a fine-pointed tenotomy knife. The clotted sinus is yellowish-white or grey, sometimes brown. It is inelastic and non-lustrous; it does not bleed profusely when punctured. The walls may be found to have sloughed, and the disintegrating clot may be characterised by the presence of minute black particles, mingled with pus. It may be difficult to determine whether thrombosis has commenced when we find a perisinous abscess and granulations on the sinus walls diminishing their normal elasticity. In deciding whether to explore the sinus by puncture we shall be guided by the clinical manifestations, which we have already considered; if the symptoms suggest the possibility of sinus infection, the sinus should be unhesitatingly explored.

Fig. 1.—Horsley's Nibbling Forceps.
(Another pattern with jaws at right angles to handles is specially recommended.)

Fig. 2.—Jansen's Nibbling Forceps.
(Also made with a double curve in the jaws.)

Fig. 3.—De Vilbiss's Bone-cutting Forceps.

Having determined that the sinus is infected, the next step is to occlude the vessel above and below the diseased area, and remove all septic material. To expose fully the sinus away from the origin of infection, the scalp is incised from the middle of the mastoid wound, along the superior curved line of the occipital bone. The pericranium having been detached over an area corresponding to the sinus, the bone is channelled with a wide gouge until dura mater is exposed. The opening is enlarged with the bone-cutting forceps until a considerable length of the sinus is in full view. The sinus is then traced backwards to a part normal in appearance, and the vessel is punctured with a narrow knife. Profuse bleeding may be taken to indicate that a non-infected portion has been opened. The sinus is at this spot plugged with fourfold gauze strips, one inch wide. After controlling the blood current above the seat of infection, we proceed to gain control below.

If it were always possible to open and occlude the sigmoid portion of the lateral sinus below the clot, exposure of the jugular vein would be unnecessary. As a rule the infected coagulum has already extended at least as far as the jugular bulb or as low as the entrance of the common facial vein, so that the usual course is to ligature the internal jugular just above the common facial vein. Wherever the ligature is applied, it must be below the thrombus. As mentioned above, a separate set of ligaturing instruments should be set aside for this part of the operation. The neck, which has been previously disinfected, should again be cleansed and guarded by sterile towels from the septic cavities opened above. The operator should also re-disinfect his hands and pay the strictest regard to aseptic principles. Probably the vein will be found less distended than is normal. Solid coagulum is recognisable to the touch. The unclotted vein is tied in two places and divided. The upper end of the vein with the contained coagulum should be dissected free, and brought out to the surface of the wound. The lower part of the incision in the neck is now sutured, while the upper end of the wound is left open for drainage. All clot is carefully detached with the curette and removed from the vein, jugular bulb, and lateral sinus. It may be necessary to expose the jugular bulb, but this procedure has not yet obtained general recognition. After the infected sinus has been laid freely open and infected coagulum removed, the wound may be gently flushed with antiseptic lotion. The meatus is enlarged by fashioning a concho-meatal flap, as in the plastic stage of the radical mastoid operation. The meatus is packed with gauze, and the post-aural wounds are also packed widely open, to secure ample surface drainage. The bleeding vessels having been ligatured, the outside dressings are applied and the patient returned to bed. The after-treatment of the wound consists in daily dressing and irrigation, and we must always be on the outlook for signs suggestive of brain abscess or of more remote collections of pus, especially empyæmata.

## Figures and Tables

**Fig. 1. f1:**
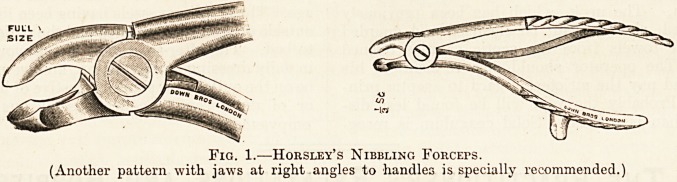


**Fig. 2. f2:**
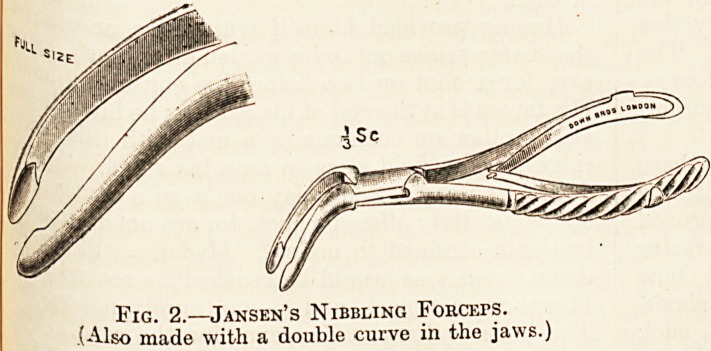


**Fig. 3. f3:**